# An ego-centred approach for the evaluation of spatial effects on health in urban areas based on parametric semi-variogram models: concept and validation

**DOI:** 10.1186/s12874-021-01300-2

**Published:** 2021-06-01

**Authors:** Odile Sauzet, Janne H. Breiding, Kim A. Zolitschka, Jürgen Breckenkamp, Oliver Razum

**Affiliations:** 1 Bielefeld School of Public Health, Bielefeld University, Bielefeld, Germany; 2 Centre for statistical consulting, Bielefeld University, Bielefeld, Germany

**Keywords:** Spatial effects on health, Spatial correlation structure of residuals, Semi-variogram

## Abstract

**Background:**

Neighbourhood is a complex structure but of high relevance for health. Its operationalisation remains however a challenge.The aim of this work is to present a new application of the use of semi-variograms as an approach for the evaluation of spatial effects on health. For this, we propose to estimate two parameters providing a measure of an average neighbourhood or spatial effect at city level without having to predefine any notion of physical neighbourhood.

**Methods:**

We present the statistical method to estimate the parameters of this correlation neighbourhood by fitting an exponential model to the empirical semi-variogram at short distances. With a simulation study, we show for which sample size and sampling density the method performs well and illustrate how to use the method with data from a birth cohort using the outcome birthweight.

**Results:**

For small sample sizes (500) the method provides reliable estimates if the density of observations is high. For larger sample sizes other parameters influencing the quality of estimates are the maximal distance at which the semi-variograms are estimated.

**Conclusions:**

Given the complexity of spatial scales relative to neighbourhood spatial processes, our approach offers the possibility to incorporate existing approaches to the operationalisation of neighbourhood in quantitative analyses while providing a measure of the part of health inequalities which could be possibly due to unmeasured spatial exposure as well as a measure of their spatial scale.

## Background

Neighbourhood in the context of health inequalities comprises a complex mixture of physical, structural, and social factors which cannot be simply defined by administrative boundaries. Neighbourhood is a complex structure but of high relevance for the evaluation of health inequalities. It possesses physical and social attributes for which pathways to health inequalities could be hypothesised [1]. Studying neighbourhood effects on health inequalities is a booming subject in the literature, with the number of quantitative studies increasing since Wilson published his book on inner cities poverty in 1987 [2, 3]. While important methodological improvements have been made in particular the operationalisation of factors on neighbourhood level [1], the operationalisation of the neighbourhood itself remains a challenge [2, 4]. Criticism of the current state of affairs has increased [2, 5] but no solution has so far been proposed. A reason for this may be that there is no unique definition of neighbourhood.

### Concept of neighbourhood in studies on contextual effects on health

The concepts of neighbourhood seen in the literature can be classified into two groups: those based on theory or qualitative data and those used for pragmatic reasons because of the availability of aggregated quantitative data.

Quantitative studies on neighbourhood effects on health rely mostly on multilevel modelling. These models assess the part of variability in health outcomes which can be attributed to the neighbourhood [6]. For this, neighbourhoods need to be predefined by non-crossing geographical entities that are often determined by existing administrative units (e.g. census tracks) to which observation can be linked and for which aggregated data is either available or the attribute can be evaluated. This type of definition might be relevant when the administrative entities play a role in terms of municipalities’ development or enrolment into schools. However, it is unlikely to be always the most relevant unit to the study of a particular health outcome. Some critics point out that the field needs a concept of neighbourhood based on theory [2, 4].

Chaix introduced the concept of ego-centred neighbourhood [7], also known as bespoke neighbourhood [8]. This approach assumes that the most relevant spatial scale for spatial exposure is the one close to residence. This leads to the more recent quantitative approach by defining neighbourhood based on the idea that administrative neighbourhoods are not relevant for daily activities and therefore to measure exposure. This concept can be operationalised tracking the movements of participants, i.e. their daily activity making it an ego-centred approach. This means that each participant has his/her own neighbourhood defined by various delimitations of the area visited [7].

Another approach is to assess particular spatial exposure by establishing the relevant spatial scale. Petrović et al. have used a multiscale approach to estimate the exposure to the socio-spatial context at different ego-centred radii using the example of exposure to neighbours with migration history [9]. This is possible only if routine statistics are available for very small units. Some qualitative studies have shown that from the perspective of the individual, the neighbourhood is defined first by immediate environment composed of the block in which one lives, and second where infrastructures like shops or schools, are located [10]. These neighbourhoods reflect different levels of encounters (social neighbourhoods) with frequent weak ties encounters (persons with which one has contact without being friends [11]) for the immediate neighbourhood and far less contact for the infrastructure neighbourhood.

Another theoretical notion often used in the literature is the one of perceived neighbourhood. This notion does not require to define a physical neighbourhood with boundaries. While it still consists of a physical area, it is only represented by attributes: perceived safety, perceived cohesion, etc. However, what neighbourhood means is not explicitly specified. Rather this concept of neighbourhood is individual and a perception does not need to be shared by two persons living nearby. Being easy to operationalise, relationship between perceived neighbourhood attributes and health are commonly studied.

### Small area of health inequalities

Population subgroups that are socio-economically disadvantaged have a higher risk of morbidity and of premature death, relative to those who are better off [12]. Heterogeneities are not limited to groups of populations with individual level characteristics, they extend also to the contextual level, i.e. between neighbourhoods, cities, or regions [13, 14]. This comprises heterogeneities with regard to ambient air or noise pollution emitted by road traffic, the spatial accessibility to services, the configuration of the built environment, or social processes, for instance (the lack of) collective efficacy, contagion of unhealthy behaviour, or (relative) deprivation that depends on the nature of the neighbourhood [15].

The variety of small-area characteristics within a city can have the positive effect of allowing diverse populations to flourish, but some of these factors may lead to heterogeneities in health outcomes aside from those due to individual characteristics which may be inequitable. A way to measure small-area effects on health is to examine if the health outcomes of persons living near each other are correlated even after controlling for possible compositional effects (correlation due to the correlation of individual characteristics of people living near each other). Health inequalities are not a feature of the individual rather a feature of a society as a whole.

The development of interventions and policies to reduce small-area health inequalities necessitates to know what is a relevant spatial scale for those inequalities [16] and move away from a one size fits all neighbourhood concept [17]. Knowing for example what is a relevant ego-centred spatial scale to improve physical activity allows improvements in the built environment which is relevant for this outcome.

The aim of this work is to present a new approach to evaluate spatial effects on health outcomes in particular the presence of contextual health inequalities in an urban setting. With spatial effects we mean all measured or unmeasured effects related to the place of residence whether they are due to the composition of the neighbourhood (e.g. socio-economic status of other inhabitants) or due to the place itself (e.g. noise or air pollution). The latter are usually called neighbourhood effects. The approach presented here is a new application of existing methods more commonly used in ecology or agriculture. Analogue to assessing the relationship between soil and growth of plants by modelling the spatial correlation structure of plant growth, considering the correlation structure based on the Euclidean distance between persons living in an urban area to measure effect of the urban setting might be relevant due the high density of the population. This approach loses its relevance when considering rural populations, however, because the large physical distance between "neighbours" means that the actual location point of one person has no relevance for the nearest living neighbour. Unlike in the usual analysis of spatial correlation for health outcomes, the unit of observation is the individual and therefore the geo-location of places of residence must be available.

An ego-centred approach to modelling the spatial correlation structure of health outcomes consists of fitting a parametric model to a semi-variogram. This provides two parameters to define an average ego-centred (individual) neighbourhood (radius and strength of the structure). In the following, we will present the method and the results of a simulation study aimed at showing the reliability of the method for sparse observational data of different sample sizes and densities. Then we illustrate the method with data on birthweight from the “Gesundheit von Babys und Kindern in Bielefeld” (BaBi) birth cohort [18].

## Methods

### Concept

Our conceptual approach is ego-centred and based on the spatial correlation of health outcomes. We measure how much the health outcome of a person correlates the health outcomes of her/his neighbours on average over a city. This approach neither necessitate the definition of boundaries nor an a priori definition of neighbourhood. Still, it allows to measure spatial effects on health outcomes if a spatial correlation structure remains after individual characteristics have been accounted for. To achieve this, we obtain a parametric characterisation of the spatial correlation structure. The correlation neighbourhood can be statistically approached using a parametric model for the so-called semi-variogram [19], a method commonly used in ecology and agriculture. The semi-variogram is a way to model the spatial correlation structure between (health) outcomes collected from geo-located observations and provides an estimate for the distance H (the practical range) such that two persons separated by a distance greater than H will have, on average, practically uncorrelated health outcomes. Also a measure of the strength of the correlation known as relative structure variability (RSV) is obtained by taking the ratio between the part of the total variance which is spatially structured (see below for more details). People with similarities tend to live close to each other: income is a factor that predicts whether one lives in a affluent part of a town, or the presence of a large ethnic community could encourage people of the same ethnic origin to move to a certain area. This means that individual characteristics possibly show a strong spatial correlation structure, which in turn could explain a spatial correlation of health outcome if these characteristics are predictors of health. Using a regression analysis allows to model the correlation structure of the part of the outcomes which is not explained by observed individual characteristics. The residuals of a correlation model will themselves show a spatial correlation structure if the variables in the model do not explain all of the spatially structured variability in the data. This approach allows to incorporate existing methods for the evaluation of neighbourhood effects.

### Statistical model

Under certain conditions of the underlying stochastic model which we will not show in detail here (the outcome is generated from a second order stationary random field with the consequence that the correlation between two points depends only on the Euclidean distance between these two points, see [19], Chap 4, Section 2), the correlation *C*(*h*) between the health outcome *Z*(*s*) at the observation point *s* and the health outcome *Z*(*s*^′^) at the observation point *s*^′^ can be assumed to be only dependent on the distance (lag) *h*=||*s*^′^−*s*|| between the two observations. For this, a constant variance of the health outcome is assumed over the whole surface where the correlation structure is being modelled. Parameters describing the spatial structure of the correlation function *C*(*h*) can be estimated using a parametric model for the semi-variogram *γ*(*h*) defined for a lag *h* as

$$\gamma(h)=\frac{1}{2}Var[Z(s)-Z(s+h)] $$

which can be estimated from the data. The empirical semi-variogram *γ*(*h*) and the correlation function *C*(*h*) have under the second order stationary assumption the following relationship:

$$C(h)=c_{0}+\sigma^{2}_{0}-\gamma(h) $$

where $\sigma ^{2}_{0}+c_{0}=Var[Z(s)]$ is the variance of the health outcome assumed to be constant everywhere over the surface studied and *c*_0_=*γ*(0), called the nugget effect, is the value of the semi-variogram when the distance between two observations tend to 0. Then $\sigma _{0}^{2}=C(0)$, the asymptotic correlation between two observation on the same point, is the so-called partial sill. An unbiased estimate (Matheron’s estimator) for the empirical semi-variogram at distance h between two observations is given by [20]

$$\hat\gamma(h)=\frac 1 {2|N(h)|} \sum_{(s_{i},s_{j})\in N(h))}\{Z(s_{i})-Z(s_{j})\} $$

where *N*(*h*) is the set of all observations lagging at distance *h*. In practice lag intervals are used (bins). A parametric model can be fitted to the estimated semi-variogram function from which we can obtain the so called practical range: the distance H above which the correlation between two observations is less than 5% of the total variance. This model (exponential) is the following:

$$\hat{\gamma}(h)=\hat{c}_{0}+\hat{\sigma}_{0}^{2}(1-\exp(-\hat{\phi}h)). $$

The practical range for this model is given by

$$H=\frac{1}{\hat{\phi}} \log \left (\frac{\hat{\sigma}_{0}^{2}}{0.05(\hat{c}_{0}+\hat{\sigma}_{0}^{2})}\right) $$

A measure of the proportion of the total variance with a spatial structure is given by the relative structured variability (RSV): partial sill/total variance which described the degree of spatial structure:

$$RSV=\frac{\hat{\sigma_{0}}^{2}}{\hat{\sigma_{0}}^{2}+\hat{c_{0}}} $$

The practical range and partial sill from an exponential model are in effect estimated from a empirical semi-variogram. Practical range and partial sill depend on the data available but also on the choice of bins in which semi-variogram points are estimated. The shape of the semi-variogram depends on the maximal distance at which the semi-variogram is estimated. The goodness of fit of an exponential model to fit the semi-variogram can be made visually.

### Simulations study: method

The aim of following simulation study is to establish how the method can be used in practice and provides recommendations about practicable sample sizes and sampling design (density of observations). The simulation were formed using *R* version 3.4.3 [21].

#### Data simulation

We simulate a complete population living in a grid "city" of 1000^2^ units. A total number of 250 thousand points were generated by using the runif function with minimal value 0 and maximal value 1000. Then, a correlation structure was generated with RMexp(var=40, scale=6) + RMnugget(var=60) and added to the generated points with the RFsimulate function of the package RandomFields [22].

#### Sampling method

First, the generated points (see “Data simulation” section) were divided into a rectangular grid with a total number of *K*^2^ grids. A two-staged sampling method was chosen. The first stage involved a probability proportional to the size (PPS) design with no replacement in which a number of grids were sampled, and the second stage was a random sampling with no replacement in which points were drawn from the sampled grids into the subsample. The choice of a PPS design and a subsequent random sampling ensured that each point of the total data set had the same probability of being drawn into the sub-sample when *n*_*k*_>>*n*_*k*,*sub*_ (i.e. sampling points without replacement can be treated as sampling points with replacement) with *k*∈{1,...,*n*_*k*_}, *n*_*k*_ being the number of points in the k-th grid and *n*_*k*,*sub*_ being the number of drawn points from the k-th grid.

The following shows which parameters were considered for the partial sample selection:

- subsample size: 500,1000,5000,9500
- total number of grids: *n*_*k*_=1,4,9,16,25
- number of grids for sampling: *n*_*k*,*sub*_=1,...,*n*_*k*_.

#### Variogram parameter estimation

For each parameter composition, the sampled points were first converted into geodata using the as.geodata function from the package geoR [23]. Variograms were then computed by using the variog function of the geoR package with the arguments

estimator.type=’classical’ and max.dist={20,40,60,80,100 },

meaning that a total of 5 variograms were computed for each parameter combination. With these variograms, the parameters were estimated using the variofit function of the geoR package with the arguments

ini.cov.pars=c(40+60,6) and cov.model=’exponential’.

#### Simulation

With all the above mentioned parameters (see Sampling method and Variogram parameter estimation) a total number of 440 parameter combinations was reached. The sampling and semi-variogram parameter estimation was repeated 500 times for each parameter combination. For comparison, a simulation study for no spatial correlation by setting

RMexp(var=0, scale=6) + RMnugget(var=60)

in Data simulation and

ini.cov.pars=c(60,6)

in Variogram parameter estimation was also performed.

The reliability of the method was assessed by the percentage of datasets for which the estimation algorithm did provide invalid results (difference between estimated total variance and sample variance larger than 10% of the sample variance), bias, mean square error and coverage of the 95 confidence interval. Results were presented in terms of sample size, density and maximal distance used to obtain the estimates.

### BaBi study

We illustrate the concept of the correlation neighbourhood by modelling the spatial correlation structure of the birthweights from the BaBi (Gesundheit von Babys und Kindern in Bielefeld) birth cohort [18]. Over a three-year period (2013-16), 977 pregnant women or women who just gave birth were recruited in three hospitals or in gynaecologists/midwives practices in Bielefeld, North Rhine-Westphalia, Germany. Informed written consent from all the participants was obtained for the interviews and access to their medical records. The study protocol was approved by the ethical committee of the Medical Faculty of Muenster University and the Data Protection Board of Bielefeld University. Using the addresses provided by the participants, we obtained the geo-coordinates which were subsequently geo-masked in order to anonymise the data. We modelled the spatial correlation structure of (non-adjusted) birthweights. Then using the student residuals of a linear regression model in which birthweight is regressed on gestational age, gravitas (first pregnancy vs. subsequent pregnancies), body mass index (BMI), income and age of the mother, we obtained adjusted estimates of the parameters of the correlation neighbourhood. The aim of the analysis was to see if there is an indication of spatial effects associated with birthweight and then to evaluate the relevant spatial scale for this effect, if it exists.

## Results

### Simulation study

**Fig. 1:**
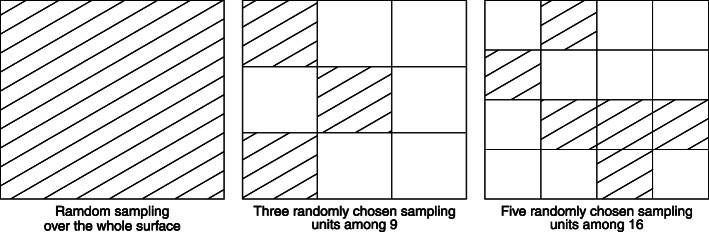
Three examples of possible sampling area from the simulations scenarios

**Fig. 2:**
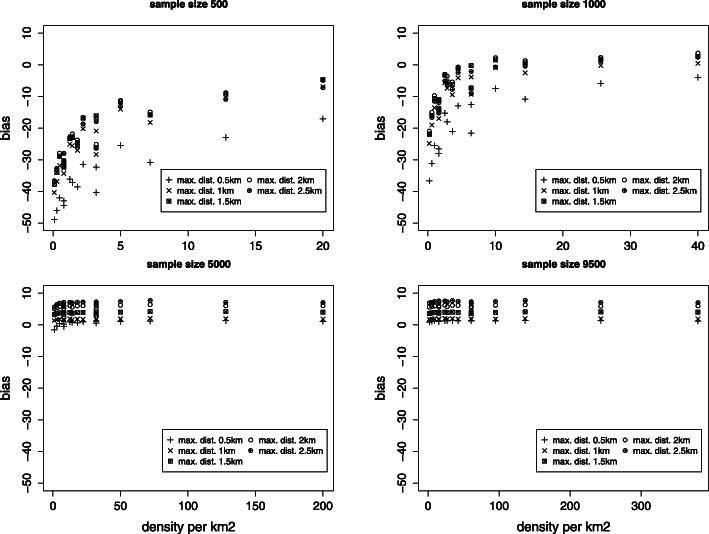
Absolute bias for the nugget effect *τ*^2^ (true value *τ*^2^=60). Rates among valid results

**Fig. 3:**
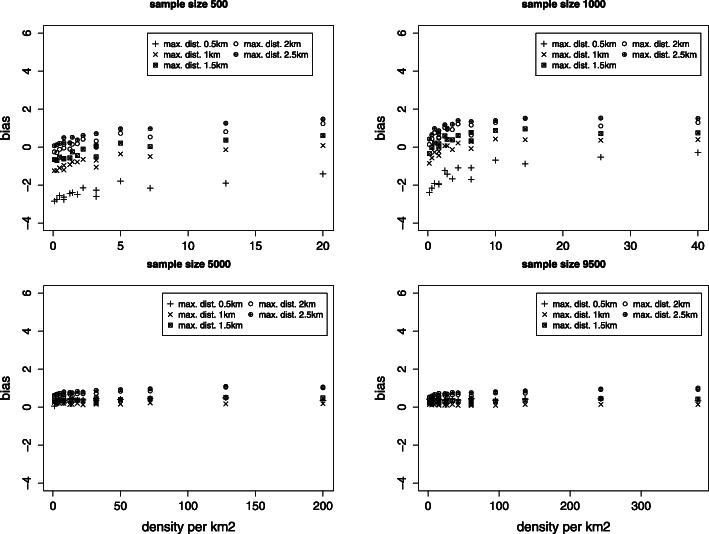
Absolute bias for the shape parameter *ϕ* (true value *ϕ*=6). Rates among valid results

**Fig. 4:**
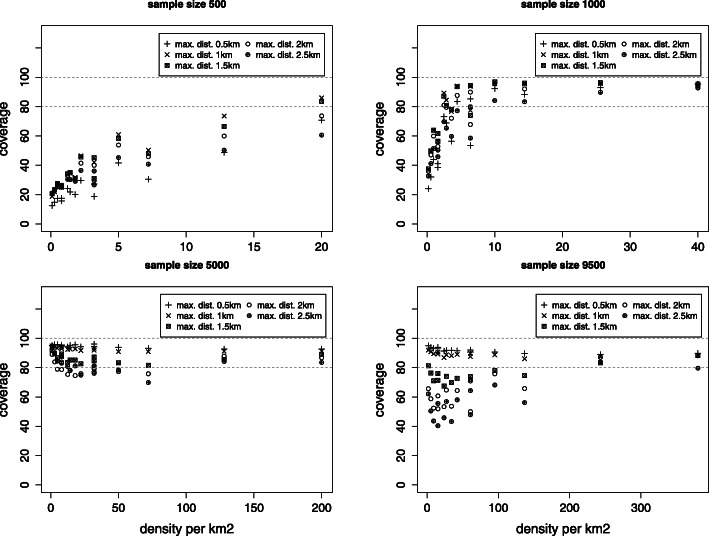
Coverage of the 95% confidence interval for the nugget effect *c*_0_

**Fig. 5:**
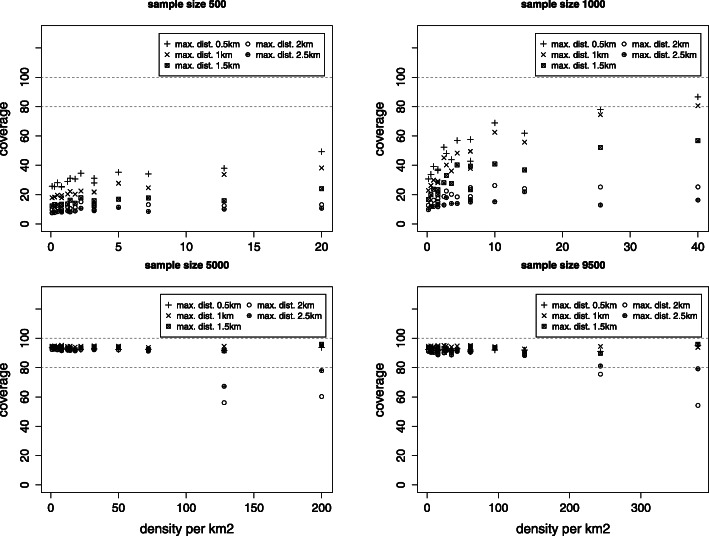
Coverage of the 95% confidence interval for the shape parameter *ϕ*

The aim of the simulation study was to assess how the correlation neighbourhood method would work for the type of data collected in social epidemiology. The results of the simulation are provided in Figs. 2, 3, 4, and 5. The sampling procedure was designed so that different degrees of clustering were achieved (see examples in Fig. 1). The sampled points belonged to a various numbers of sampled grids units of various sizes. This way we obtained a range of densities and a range of number of clusters. Because density (and not the number of clusters) was the most relevant factor to determine the reliability of the method, the results are presented in terms of density of observations per km^2^.

Factors determining the reliability of the estimates include the maximal distance between observations used for the estimation of the empirical semi-variogram as it determines the shape and the precision of each point, the global sample size, and the density of observed points within the selected areas.

#### Non-estimated cases

Here we include all simulated samples for which at least one of the estimated exponential model parameters was improbable, indicating that the algorithm did not converge. This is mostly a problem for the sample size of 500 where the rate of non-convergence does not go much below 20%. The rate of non-convergence decreases with density of observations and varies with maximal distance. When non-convergence occurs it is advisable to change the maximal distance or the number of bins used to estimate the empirical semi-variogram.

With a sample size of 1000 or more also for low sampling density, the rates of non estimation can be well under 20% and decrease with increasing maximal distance. For larger sample sizes even with small densities the algorithm will always converge, if the maximal distance is large enough.

#### Bias

Considering only cases where the algorithm did converge, the bias for the overall variance remains for all sample sizes and maximal distances less than 1% with the exception of the smallest maximal distance and a sample size of 500. Non-biased estimates of partial sills and nuggets (Fig. 2) are obtained for the smallest maximal distance and a sample size of 5000 and more. For smaller sample size the density plays a major role on bias, indicating that sampling only a small number of areas is important if the sample size is small. The estimation of the scale parameter (which provides the range) depends strongly on the maximal distance used. For larger sample size the bias remains small (Fig. 3).

The role the maximal distance plays on the bias of estimates is important. Some bias can be induced using a too large maximal distance as seen for large sample sizes. Hence it is recommended to reduce the maximal distance so long as the sill is still reached.

#### Mean squared error and coverage of the 95% confidence interval

The standard error for all estimated parameters is obtained as the standard deviation over all simulations for a given scenario. MSE estimates show that the standard errors depend not just on the sample size but also on the density and the maximal distance. The coverage of the 95% confidence interval is near 95% for the smallest maximal distance and there are indications that, when the density is large enough, the coverage can be acceptable even for smaller sample sizes (Figs. 4 and 5).

#### Type i error

Data were also simulated with a nugget effect equal to the total variance in order to check the probability to see a spatial correlation structure which did not exist. The rate of non convergence was between 18 and 25% for all sample sizes and density. Only for a sample size of 500 and the lowest density it is possible to conclude that a spatial correlation structure exists. A mean nugget effect of up to 10 was found. However for larger densities or sample sizes the mean nugget did not exceed 1 (compared to a variance of 60).

#### Summary

The reliability of the method depends on both the sample size and the density of observations on the sampled geographical areas. In other words if the sample size is small (<1000), the method remains reliable if the observations are close to each other. Another element of reliability is the maximal distance used for the estimation of the parameters. It is therefore important to fit the exponential model to a range of maximal distances and choose the one for which the best fit is obtained. The reliability of the estimates can be checked by visualising how well the estimated curves fit the data and how much the estimated total variance (nugget plus partial sill) deviates from the sample variance.

### Spatial correlation structure of birthweight data

**Fig. 6:**
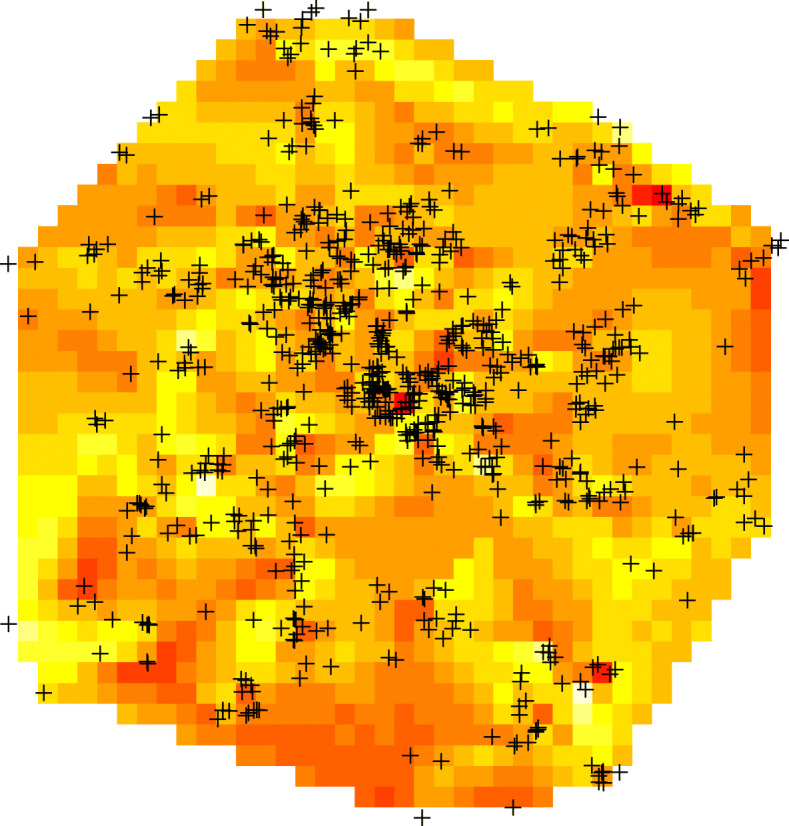
Representation of the BaBi data using interpolation with geo-coded location of observation. Birthweight increases with darkness of the representation. Scale: x-axes 15 km, y-axis 20 km

The participants in the BaBi Study were living less than 100m away from an average of 1.7 fellow participants with numbers ranging from 0 to 6. Looking at a distance of 200 m, this increased to an average of 3.3 participants with numbers ranging from 0 to 15. In terms of average density we can consider how many fellow participants are found in a radius of 564m (Surface of one km2). On average 13 participants lived within this radius ranging from 1 to 52. With just under 1000 observations, according to our simulation study, the method should provide reliable estimates of the parameters of the correlation neighbourhood. Figure 6 provides a spatial representation of the birthweight data using interpolation (R package akima [24]). The mean birthweight was 3 400 g with standard deviation 473 ranging from 970 to 4 830 g.

**Fig. 7:**
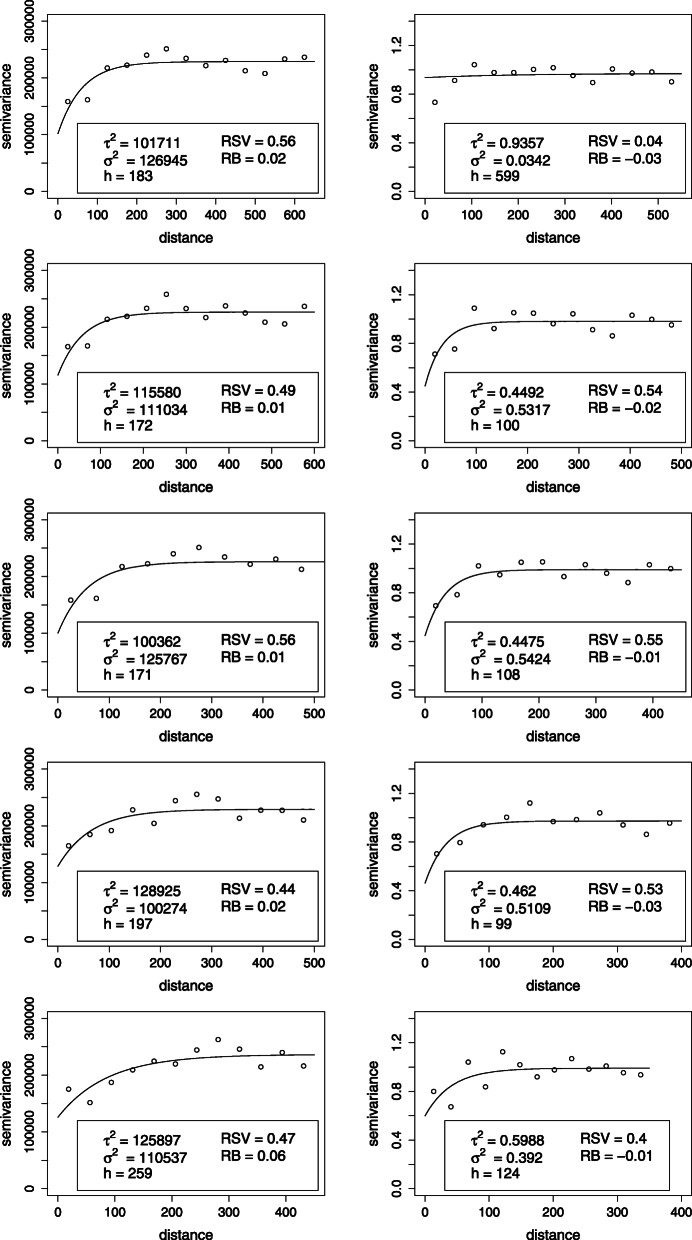
Empirical semi-variograms and fitted exponential models for different maximal distances for birthweight (left) and birthweight adjusted for gestational age, BMI, gravida, and income

After estimating the semi-variogram for several maximal distances (350 to 650 m) and fitting exponential models, the best maximal distance to detect an exponential structure for the spatial correlation of birthweight was 500m for a semi-variogram estimated over 11 lag intervals (See Fig. 7). We obtained an estimated nugget effect of 3552 and a partial sill of 3222 providing a RSV of 44%. The total estimated variance was 2% higher than the sample variance of birthweight. The range was 197m (see Table 1)

**Table 1:** Parameters of the exponential semi-variogram for birthweight data for varying maximal distances (bold face: best fitting model)

Birthweight				Residuals ^∗^			
Max. distance	Range	RSV	Bias ^∗∗^	Max. distance	Range	RSV	Bias ^∗∗^
650 m	183 m	0.56	0.02	550 m	599 m	0.04	-0.03
600 m	172 m	0.49	0.01	500 m	100 m	0.54	-0.02
550 m	171 m	0.56	0.01	450 m	108 m	0.55	-0.01
**500 m**	**197**	**0.44**	**0.02**	400 m	99 m	0.53	-0.03
450 m	259 m	0.47	0.06	**350 m**	**124 m**	**0.40**	**-0.01**

^∗^ Residual after adjusting for gestational age, BMI, gravida, and income. ^∗∗^ Relative bias: (partial sill+ nugget - variance)/variance

In a further step we regressed birthweight on gestational age, BMI of the mother, gravida and income and fitted an exponential model to the semi-variogram for the Student residuals of the linear regression model using the same maximal distances as for the raw birthweight. The results are presented in the left column of Fig. 5 for maximal distances ranging from 350 to 550m. The best maximal distance to detect an exponential structure for the spatial correlation of the residuals was 350m for a semi-variogram estimated over 13 lag intervals (See Fig. 7). We obtained an estimated nugget effect of 0.60 and a partial sill of 0.40 providing a RSV of 40%. The estimated total variance was 1% lower than the standardised residual variance of 1 with a range of 124m. In order to evaluate the validity of the exponential model we need to observe the fit of the exponential model to the estimated semi-variogram in Fig. 5. For both the unadjusted and adjusted models, the estimate for the range is adequate while a high variability for small distances between observations indicate that the nugget effects might be underestimated. However an exponential model fits the data well showing that birthweights are spatially correlated. After adjusting for some socio-demographic factors, we obtained a correlation structure for the residuals which showed a weaker structure than the raw data: a reduced maximal distance and a smaller RSV. This means that a smaller part of the total variance is spatially structured and that the effect of one location on birthweight is less far reaching. This occurs because some (but not all) of the spatial correlation seen for birthweight is due to the spatial correlation structure of the covariates we adjusted for. In particular income tends to be spatially correlated due in particular to house prices or desirability of an area.

## Discussion

In this article we have introduced the use of semi-variograms to assess the presence of spatial effects on health without having to specify any particular geographical units to define neighbourhoods. Using simulated and real data we have explained and illustrated the potential of modelling the characteristics of the spatial correlation structure over a city. Because this approach provides two parameters to measure neighbourhood/spatial effects on health inequalities and offer a measure of scale for these effects, we use the term “correlation neighbourhood”.

Small area health inequalities occur if the factors related to the place of residence affect health outcomes independently of individual circumstances and it is assumed that if such effects exist, then the health outcome of neighbours will be correlated through spatial effects of place. However this correlation decreases when the distance between neighbours increases. Thus the characteristics of the spatial correlation structure of health outcomes provide an indicator of the presence of small-area health inequalities.

Modelling the spatial correlation structure of a health outcome provides a measure of how much a health outcome is correlated to the health of one’s neighbours. It is a way to assess if and how much the health outcome tend to be clustered (potentially meaning strong health inequalities due to the context of residence) or weakly correlated (indicating that there might be little health inequalities due to the context of residence). The random variance estimated using multilevel models is a measure of unobserved factors within predefined geographical units [6]. But an advantage of the parameters of the correlation neighbourhood is that they provide measures of intensity of the spatial effects and of a scale on average over a city.

### The method in practice

As seen in the simulation study, it is important to fit exponential models for semi-variograms estimated on the basis of different maximal distances or numbers of bins. Then compare the differences between the estimated total variance (partial sill plus nugget) with the sample variance to obtain the best goodness of fit. Visual checks should also be performed to see how well the model fits the semi-variogram. Like the multilevel approach to assess unmeasured spatial effects, the correlation neighbourhood approach has so far a descriptive character. Hypothesis testing or obtaining confidence intervals for semi-variograms is difficult and not reliable in the context of sparse non-experimental data. In case of difficulties with the estimation one can either change the size of the bins to increase the number of pairs of observations within the lag intervals or increase the maximum distance at which the semi-variogram is estimated (thus automatically increasing the number of observation per bin). The parameters obtained depend on the bins chosen to estimate the semi-variogram. If the estimated semi-variogram is not smooth, the parameters obtained for the exponential semi-variogram from various distances may be substantially different. In this case the model obtained may be inadequate. It is of little relevance to model only the correlation structure of raw outcomes as it will merely show that individual predictors of health outcomes are spatially correlated. In practice, the spatial correlation of residuals of regression models should be modelled. Hence, the correlation neighbourhood approach is an additional tool to the existing methodologies available to assess spatial or neighbourhood effects - including multilevel models to account for predictors obtained for administrative units (e.g. unemployment rates).

The sample size and density of observational health data is usually limited. Our simulations have shown that an important factor for sampling scheme would be to optimise the density of observations to improve the reliability of the method if the sample size is limited. However we have shown that the method works reliably with sample size of 500 if the density is sufficient.

## Conclusion

Given the complexity of spatial scales relative to neighbourhood exposure [25] our approach offers the possibility to incorporate existing approaches to the operationalisation of neighbourhood in quantitative analyses while providing a measure of the part of health inequalities that may be due to unmeasured spatial exposure. Moreover, the method provides a measure of small-area health inequalities.

## Data Availability

Data was used for illustration purposes. Data are available upon request due to ethical restrictions. Interested researchers may submit requests to Dr. Céline Miani, leader of the BaBi Study, School of Public Health, Bielefeld University (Contact: Universitätsstraße 25, 33615 Bielefeld, Germany. E-mail: celine.miani@uni-bielefeld.de), or to Mrs. Anja Schmid, data protection and data security officer, Bielefeld University (Contact: Universitätsstraße 25, 33615 Bielefeld, Germany. E-mail: anja.schmid@uni-bielefeld.de). Declarations

## Abbreviations

BMI: Body mass index
